# Income inequality, consumption, and the debt ratio of Chinese households

**DOI:** 10.1371/journal.pone.0265851

**Published:** 2022-05-11

**Authors:** Zhaolin Shen, Wei Fan, Jiang Hu

**Affiliations:** 1 Economics and Management School, Wuhan University, Wuhan, China; 2 Business School, Zhengzhou University, Zhengzhou, China; 3 Economics and Management School, Hubei University of Automotive Technology, Shiyan, China; The Bucharest University of Economic Studies, ROMANIA

## Abstract

The increasing family leverage and the expansion of income inequality have aroused widespread concern in society. Based on the data from the China Household Finance Survey (CFPS), this paper constructs the Gini coefficient and household credit index, and aims to research the impact of income inequality, borrowing level on consumption in different regions, urban and rural areas. The results indicate that the increasing of income inequality significantly restrained the increase of household consumption, while the moderate increase of household leverage can promote household consumption to a certain extent. According to the conclusion of this article, in order to realize the sustainable growth of consumption and upgrade of structure, the government should take measures to stimulate domestic demand: (1) For the central and western regions, the overall wealth level of households should be improved and the borrowing level of residents should be appropriately increased; (2) For the eastern region, the income inequality should be controlled and reduced the negative effects of income inequality on sustainable consumption growth. This research provides evidence for understanding the relationship between income inequality, household leverage ratio and consumption, and sheds light on the formulation of related policies.

## Introduction

China’s economy has experienced rapidly growth since the reform and opening-up began forty years ago, and China has become a middle-income country. With China’s economy has entered a period of new normal (the new normal period is China’s economy has entered a period of new normal, which brings an opportunity for the development of a green and low-carbon economy and environmental protection.), which brings a series of internal and external factors, including the demographic dividend attenuation, the accumulation of risks of the "middle-income trap" and the adjustment of the international economic pattern. Since 2015, the correlation between major economic indicators in China has deviated, and economic growth continued to decline. At the same time, household income has increased but corporate profits have declined, and consumption has risen while investment has declined. In order to better adapt to the changing society, China has promoted supply-side structural reform with structural adjustment and transformation of growth drivers as the main task. The priority task is to deleverage state-owned enterprises (SOEs), local governments, and financial institutions, which are currently underleveraged for the broader household sector. And on the contrary, in order to shift the economy from investment- and external-led growth to endogenous consumption-led growth, households need to be leveraged.

**Fig 1:**
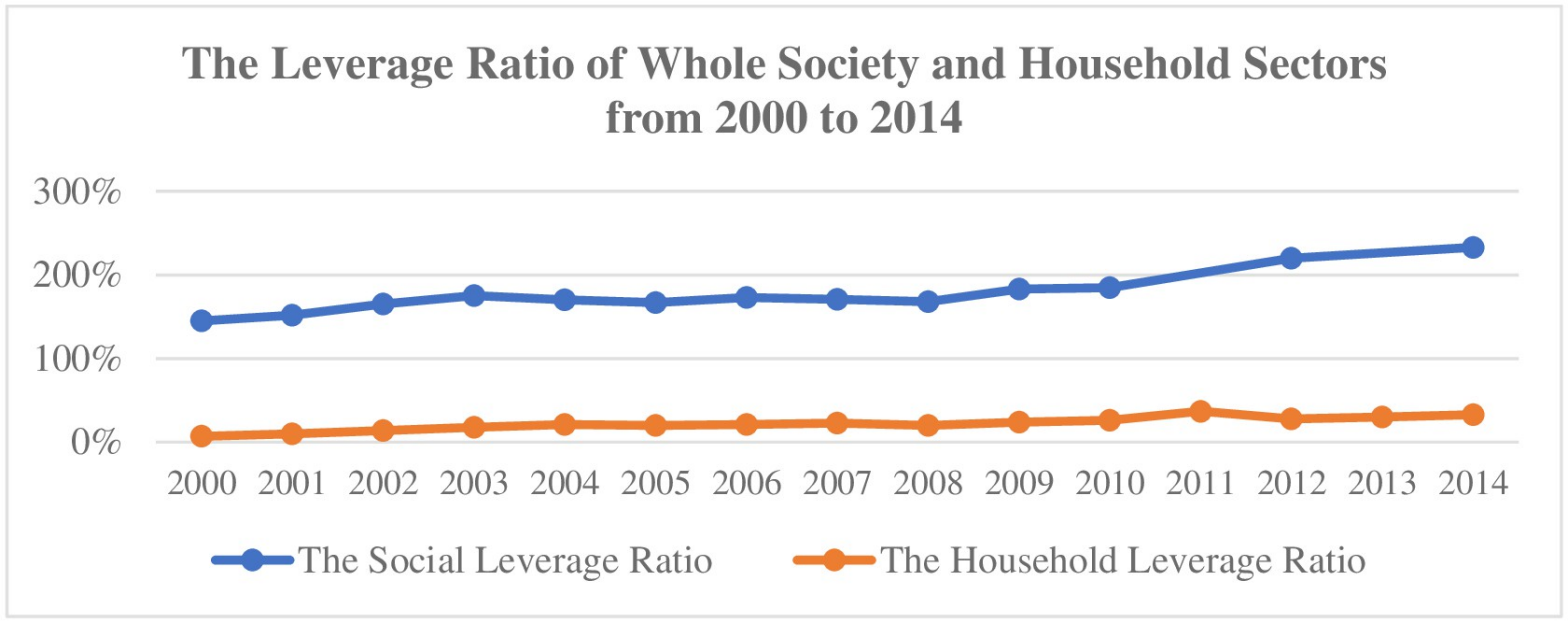
The leverage ratio of whole society and household sectors from 2000 to 2014. Source: China Statistical Yearbook.

In fact, comparing with the rapid rise of whole society leverage ratio, the absolute value and the rising speed of the leverage ratio of household sector are not too high, although the leverage ratio of household sector has increased since 2000 (see Fig 1). Cecchetti et al. [1] based on the calculation of leverage ratio data of various economic sectors in 18 OECD countries from 1980 to 2010, it shows that the impact of leverage ratio of different economic sectors on economic growth has a threshold from positive effect to negative effect. When the leverage ratio of household sector reaches 85%, it may have a negative impact on the economy. According to this calculation, China’s household leverage ratio is still stay in a relatively low position, but the income gap is expanding. Under this background, improving the leverage ratio of the household sector may promote consumption and promote the transformation of the driving force of economic growth.

Over the same period, income inequality among Chinese households is measured by the Gini coefficient, which rose from 0.42 in 2000 to 0.49 in 2010. Although it slightly decreased to 0.47 in 2015, China’s household income inequality is still among the world’s worst (Gan et al. [2]). According to the World Inequality Database from 1994 to 2015, the share of the highest income 10% and the highest wealth 10% in total income and total wealth increased, while the share of the lowest income 50% and 50% in total income and total wealth decreased. The widening of income gap and wealth gap will affect the balanced development of our society. With the accumulation of debt, asset prices rise faster than income growth brings a high risk of bankruptcy to the ultra-indebted households, which shocks financial market confidence and penalizes financial repression, further constraining credit for most households (Mian and Sufi [3]). Then, under the current economic structure of widening family income gap and income inequality in China, will family leverage bring consumption growth? How will the expansion of income inequality and the increase of household debt affect residents’ consumption behavior? How to use consumer finance to stimulate consumption, improve China’s consumption quality and unreasonable economic structure, and realize the sustainable growth of consumption? Therefore, the research on the relationship between income inequality, household debt and household consumption has become a key problem that scholars and policymakers urgently need to solve.

This paper argues that the cross of income inequality and household debt is the fundamental reason for the heterogeneity of their effects on consumption. Most of the domestic and foreign scholars only focus on the impact of income inequality on consumption or the impact of household debt on consumption, but rarely on the cross-effects of both, and lack of regional comparisons and studies on the sustainability of consumption. In view of the unbalanced economic development, the rapid rise of house prices and the stagnant consumption growth trend, this paper intends to start from the perspective of income inequality and use the family statistics from China Family Panel Studies database (CFPS) from 2010 to 2016 as the sample. Using a two-way fixed effect model of control time and province to analyze the influence of income inequality and household debt on household consumption. The research finds that household consumption is negatively correlated with income inequality and positively correlated with household debt, The decrease of income inequality will significantly enhance the positive impact of household debt on household consumption, which is applicable to both urban and rural residents. However, there is a big difference between the effects of income inequality and household debt on the central and western regions. The increase of income inequality can promote the total household consumption in the Middle region, while for the western region, the expansion of income inequality can inhibit the impact of total consumption. In addition, appropriately increasing household debt can reduce income inequality to hinder the decline of household expenditure, but this effect tends to weaken with the excessive household debt.

The rest of the paper is organized as follows: Section 2 reviews literature on the impact of income inequality on household consumption and the impact of household debt on household consumption, and Section 3 introduces the theoretical model, data and hypotheses. Section 4 presents the empirical results and analysis and, finally, Section 5 is conclusions and policy recommendations.

## Literature review

Income inequality and household consumption has become a universal concern among academia, politics and societies in recent years. Most of the early studies on the impact of widening income inequality on residents’ consumption activities were based on theoretical analyses and had different findings. A review of the existing literature suggests that widening income inequality has both positive and inhibiting effects on household consumption. Mainstream consumption theories such as Modigliani’s [4] life cycle hypothesis and Friedman’s [5] permanent income hypothesis and Hall’s rational expectation life cycle hypothesis argue that income redistribution would not affect aggregate consumption. Widening income inequality would be thought of as temporary income fluctuations, and households will smooth consumption by increasing precautionary savings, thus increasing income inequality acts as a disincentive to consumption. Zhang et al. [6] used new Keynesian dynamic stochastic general equilibrium (NK-DSGE) model to study the impact of the COVID-19 pandemic on the sustainability of Chinese economic growth and found that the monetary policy tools to alleviate unemployment and alleviate social inequality can improve the sustainability of the Chinese economy. Some domestic scholars also base on Keynes’ [7] absolute income hypothesis, which argues that household saving rate and income level are positively correlated, and thus an increase in income inequality makes the overall social saving rate increase [8]. Fan et al. [9] also argue that widening income inequality makes people more likely to pursue social status and increase human capital investment, which in turn reduces household consumption. However, in the wake of the financial crisis in the United States in 2007, many empirical studies have shown that the change in the distribution of income due to the widening income inequality, rather than a temporary income fluctuation, which has aroused the attention of the academic community to the relative income hypothesis. The relative income hypothesis was first proposed by Duesenberry [10], which argues that total household saving is not determined by the absolute value of household income, but by the consumption behavior of people around it. The reason is that households with lower relative income will increase their own consumption rate to catch up with the consumption level of higher income households to climb up the ladder, thus increasing the household consumption. Using Urban Household Survey data (UHS), Ji [11] finds that income inequality does have a negative effect on household consumption, but this effect decreases significantly after considering average regional income, mainly because the Gini coefficient is negatively related to average regional income. Hassan et al. [12] theorizes that income inequality affects household consumption through household borrowing, which can increase household consumption in the short term, but undermine sustainable growth in consumption. To investigate wealth and income inequality, Tian and Liu [13] proposed an inhomogeneous agent-based model where a large number of individual persons (agents) work, consume and invest, and it was revealed that capital (investment) income plays an essential role in producing inequality. Chatterjee et al. [14] study the distributional features and inequality of consumption expenditure across India, for different states, castes, religion and urban–rural divide, they find that state-wise differences in inequality may arise from the differences in average incomes, but consumption inequality is lower than income. In the research of China, based on the data of 2010, 2012 and 2014, Hang and Yu [15] found that the stimulation of the housing demand is more obvious than that of the housing price increase, while the income inequality has both pull and control effect on the sustainable development of urban household consumption.

Household debt is also a hot topic in recent years, and whether household leverage could promote consumption is still uncertain. On the one hand, household debt of moderate size can produce ‘wealth effect’ by increasing household leverage. With the increase of household leverage ratio, the growing household’s asset value will increase household total expenditure, and at the same time, the related industries consumption booming [16–18]. The reason for this result may be related to the diminishing marginal propensity to consume. In addition, housing prices are also an important factor. As the house prices rise, the increasing of property wealth will add household consumption by homeowners, but it will have little effect on the consumption behavior of young renters [19, 20]. Using the data of household expenditure survey from 1983 to 1989, Maki [21] found that the consumption of households owning stocks and other financial assets will change when the share price changes, but household consumption without stocks and other financial assets will not be affected, which further confirms the existence of the wealth effect. Meanwhile, Yi and Zhou [22] find that the development of inclusive finance can promote residents’ consumption, and this promotion effect is more obvious in rural areas, central and western regions, and households in the middle and lower income classes. This type of consumer finance has promoted resident consumption mainly through the two mechanisms of easing liquidity restriction and facilitating resident payment. On the other hand, due to the long mortgage liquidity cycle in the real estate market, households’ cash flow decreases because of high leverage and the liquidity constraint reduces current consumption, thus creating a ‘crowding-out effect’ on consumption. Mian and Sufi [23] argue that the excessive scale of household debt will worsen household balance sheet and lead to a decline in consumption. Dynan [24] and Edelberg [25] find that household leverage as an adjustment target and its own liquidity constraints and credit constraints have a negative impact on household consumption. Pan and Jing [26] also argue that when household income growth cannot match the rate of debt expansion, it leads to a decline in household debt servicing capacity, crowding out consumption spending and causing a cascading response in other sectors of the economy. In addition, the rapid rise in household debt is a major source of risk of financial crises in economies. The empirical study finds that rising house prices are the core factor of the rapid rise in household debt in the United States and South Korea, and the 2008 U.S. subprime mortgage crisis was the primary cause of household debt accumulation [27]. When a crisis occurs, highly indebted households are at higher risk of going bust, and a chain reaction affects the financial system, leading to a devastating financial crisis.

In addition, the impact of income inequity on household liabilities is also taken into account. Iacoviello [28] further consider a dynamic general equilibrium model with heterogeneous agents to study the trend and the cyclical properties of household debt based on the study of Krusell and Smith’s [29] framework. He finds that at long-run frequencies, the behavior of household debt closely mirrors earnings inequality. Kumhof et al. [30], for example, argue that the rising income inequality has prompted low-income groups to increase their borrowing level and improve household leverage. Based on the theoretical model of Kumhof et al., Rannenberg [31] deduced that income inequality not only leads to the expansion of debt, but also reduces the interest rate in the financial market. The above theoretical research shows that the widening of income gap promotes the expansion of debt, especially the debt level of the group with lower initial endowment, and then leads to the increase of the leverage ratio of the whole household sector. Then some scholars have also explored income inequality and household indebtedness by means of empirical studies. Mansilla [32] identifies Mexican peso crisis in 1994 finds that the low-income bulk of the distribution of both income and wealth seems to be fitted by either Log-normal or Gamma distributions in general. Coibion et al. [33] find evidence that low-income households face higher credit prices and reduced access to credit as inequality increases. They argue that these patterns are consistent with inequality tilting credit supply away from low-income households and toward high-income households, which may have long-run implications for outcomes like homeownership or entrepreneurship. However, we do not go deeper here, and just focus on their effect of residential consumption.

In general, the existing literature focuses mainly on macro-level studies and rarely uses micro-survey data representing households to explore the relationship between income inequality, household leverage, and residential consumption. According to the data from 2010 to 2016 of the China Household Finance Survey (CFPS), this paper finds that the increasing of income inequality significantly restrained the increase of household consumption, while the moderate increase of household leverage can promote household consumption to a certain extent. In addition, based on the analysis of the regional and urban rural differences, the paper believes that the government should take measures to stimulate domestic demand: firstly, for the western regions, the overall wealth level of households should be improved; Secondly, for the central and eastern regions, the income inequality should be controlled and reduced the negative effects of income inequality on sustainable consumption growth. In this sense, the research of this paper complements and improves the existing literature.

## Materials and methods

### Methods and variables

To consider the impact of household debt and income inequality on household consumption expenditure in China, we set the total household expenditure as the explained variable, the Gini coefficient and household debt ratio and the cross term as the main explanatory variable, the household net income and the household net assets and other family characteristics as the controlling variable. Firstly, we examine the direct effects of household debt and income inequality on the total expenditure, and then add the cross term between household debt ratio and Gini coefficient to analyze the relationship between household debt and income inequality.

#### Direct impact of income inequality and household debt on aggregate household consumption

Referring to the models of Zhou et al. [34] and Pan et al. [26] we set the basic econometric model as follows.


$$lnexp_{jt}=\alpha +\beta_{1}Gini_{jt}+\beta_{2}lev_{jt-1}+\beta_{3}\;ln\left(income_{jt}\right)+\beta_{4}\;\text{ln}\left(asset_{jt}\right)+\gamma X_{ijt}+\varphi prov+\tau U_{t}+\varepsilon_{ijt}$$ (1)

We logicalized the data with large value in this model (total household expenditure, the household net income and the household net assets), where the explanatory variable *lnexp*_*jt*_ denotes total household expenditure (exp) over the period 2010–2016. According to the statistical classification of CFPS data, total household expenditures include residential consumption expenditures (pce), transfer expenditures (eptran), welfare expenditures (epwelf), and expenditures on home construction and purchase loans (mortage). *lnexp*_*jt*_ denotes the average consumption expenditure of households in region j and in year t, that is, the average consumption expenditure of households at the provincial level. *Gini*_*jt*_ is a measure of income inequality, indicating the magnitude of the Gini coefficient in region j. Referring to the Gini coefficient of income calculated using the net income per capita of households in the CFPS database in Zhou et al. [34], as the key explanatory variable in this paper. Here we choose to calculate the Gini coefficient using the net income per capita of households in the full sample comparable to 2010. Firstly, we use the full-sample per capita income data instead of selected samples is because the Gini coefficient as an indicator of income inequality in a region, reduction in the number of samples will lead to a bias in the results. Secondly, since 2012, the CPFS database has made a major adjustment to the family economic questionnaire, resulting in the statistical discrepancy between the 2010 and 2012 questionnaires in terms of wage income, non-agricultural operation income and transfer income. However, the content of this paper needs to use the changes of the same group of family income between different rounds from 2010 to 2016. Therefore, the use of comparable income avoids the income changes caused by different contents of questionnaire questions. Household debt in the lagged period is not affected by household consumption in the current period, avoiding endogeneity problems arising from the possible reverse causality of household consumption affecting resident household debt. Thus, we use *lev*_*jt*−1_ denotes the leverage ratio, which is the average household gearing ratio (total household responsibility/total household assets) in region j in year t-1. In order to remove the impact of some extreme values on the study, we apply a 0.5% winsorize to the leverage ratio.

At the same time, we control other factors that may affect total household expenditure. *ln*(*income*_*jt*_) denotes the logarithm of the average net household income in region j in year t. Similarly, the average household net income is calculated using the comparable household net income of 2010. *ln*(*asset*_*jt*_) denotes the average total household assets in region j in year t. Total household assets are mainly composed of land, property, financial assets, productive fixed assets, and durable goods. To be consistent with the Gini coefficient, we average total household expenditure, household assets, and net household income by province. Control variables *X*_*ijt*_ denotes household head information and household-level control variables, which include indicators of household head characteristics such as householder, gender, age and age squared, education level, marital status, health status, and indicators of household-level characteristics such as number of household members and household labor force. Since the head of household is not explicitly defined in the CFPS database, this paper refers to the "head of household" in 2010, "member of the most familiar with household finances" in 2012, and "financial respondents" in 2014–2016 as the head of household in each year. The gender of the household head is a dummy variable, assigned as 1 for men and 0 for women. Age is the age of the household head, and the squared term of the age of the household head is introduced to take into account the possible non-linear effect of the age of the household head on expenditure. The education level of the household head is measured by the number of years of education. The marital status of the household head is categorized as unmarried, married, divorced and widowed, for simplicity, we denote married as 1 and others as 0. The health status of the household head is categorized into 5 levels: very healthy as 1, healthy as 2, relatively healthy as 3, generally healthy as 4 and unhealthy as 5. Family size indicates the number of household members, including those living in the household and those who are economically connected to the outside world. For the household labor case, we assign a value of 1 to the presence of an adult unmarried male in the household and 0 to the rest. In addition, the model contains province dummy variables (*prov*) and a time dummy variable (*U*_*t*_), and *ε*_*ijt*_ is random perturbation term.

#### Indirect impact of income inequality and household debt on total household consumption

To test the indirect impact of income inequality and household debt on total household consumption, we have introduced the cross term *Gini*_jt_*lev_*jt*−1_ of income inequality and household debt on the basis of model (1) to build model (2).


$$\begin{array}{l} lnexp_{jt}=\alpha +\beta_{1}Gini_{jt}+\beta_{2}lev_{jt-1}+\kappa_{1}Gini_{jt}*lev_{jt-1}+\beta_{3}\;ln\left(income_{jt}\right)\\ +\;\beta_{4}\text{ln}\left(asset_{jt}\right)+\gamma X_{ijt}+\varphi prov+\tau U_{t}+\varepsilon_{ijt} \end{array}$$ (2)

The cross term of income inequality and household debt *Gini*_*jt*_ * *lev*_*jt*−1_ is used to measure the cross-effect of income inequality and household debt on total household consumption. If an increase (decrease) in income inequality amplifies (reduces) the effect of household debt on total household consumption, the *κ*_1_ coefficient is significant. This paper uses Gini coefficient of income as the main measure of income inequality, and also applies to other measures such as Theil index and the Mean Logarithmic Deviation (MLD) index as proxy variables to test the robustness of the empirical results.

### Materials

#### Data source

China Family Panel Studies (CFPS) is a nationally representative, biennial longitudinal survey of Chinese communities, families, and individuals launched in 2010 by the Institute of Social Science Survey (ISSS) of Peking University, China. The CFPS is designed to collect individual-, family-, and community-level longitudinal data in contemporary China. The studies focus on the economic, as well as the non-economic, wellbeing of the Chinese population, with a wealth of information covering such topics as economic activities, education outcomes, family dynamics and relationships, migration, and health. All members over age 9 in a sampled household are interviewed. These individuals constitute core members of the CFPS. As in the PSID, children of the CFPS are also considered core members of the CFPS. Theoretically, a core member can leave the study only through death. Follow-up of all core members of the CFPS is designed to take place on a yearly basis. Five provinces are chosen for initial oversampling (1600 families in each) so that regional comparisons can be made. The remainder of the CFPS sample (8000 families) is drawn from the other provinces so as to make the overall CFPS sample representative of the country through weighting (except for remote areas, noted later).

**Fig 2:**
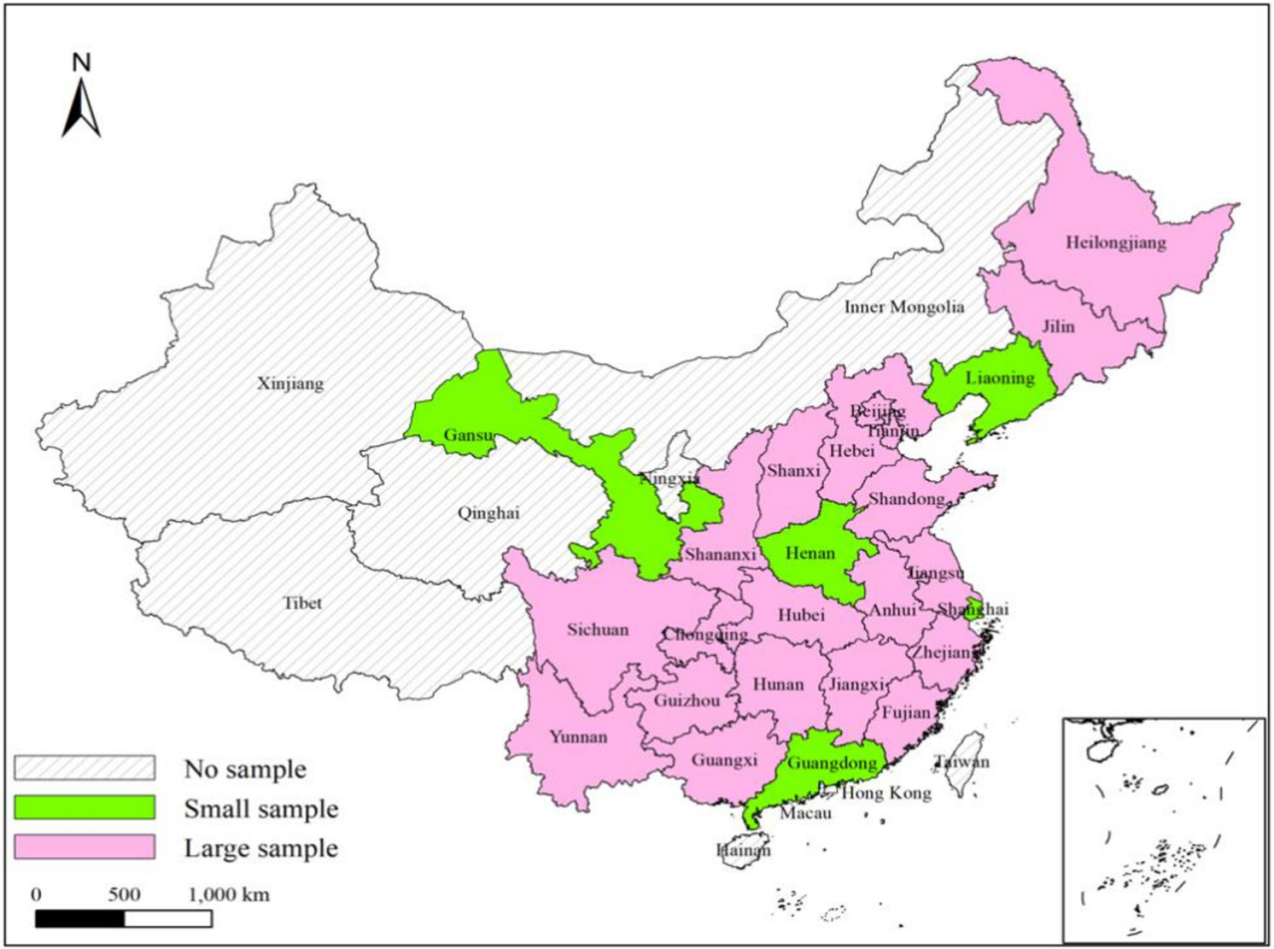
The sources of CFPS samples at the provincial level. Source: CFPS database 2010.

The CPFS (China Family Panel Studies) samples used in this paper covers the population of 25 provinces/municipalities/autonomous regions, which account for about 95% of the total population (excluding Hong Kong, Macao, and Taiwan), as shown in Fig 2, and is a nationally representative sample. Among them, the survey sample in 2010 covered 14,798 households in 162 counties (including cities) in 25 provinces across China, and follow-up visits were conducted from 2012 to 2016, respectively, with a follow-up rate of more than 70% (Zhou, 2018). The survey information of CFPS is divided into five parts, which are village questionnaire, household member questionnaire, family questionnaire, children questionnaire and adult questionnaire. The information on total household consumption, net household income, net household assets, and household demographic structure used in this paper come from the household questionnaire, while personal information such as gender, age, education level, marital status, and health status of the household head come from the adult questionnaire. In summary, this paper used household data for four consecutive years from 2010 to 2016, and 5866 households that received follow-up interviews for all four consecutive years were retained in the data collation, resulting in a valid sample size of 23464.

#### Descriptive statistics

After determining the final sample, descriptive statistics were obtained for each major variable, as shown in Table 1. As can be seen from Table 1, the number of household members in the full sample is at least 1, and the maximum number is 26, with an average of 3 to 4 persons per household. About 60% of households are headed by men, which also reflects that Chinese households are mainly headed by male members who play the role of household principals or financial decision makers. About 88% of the household heads are married, and the average age is 50 years old, but the average education level is not high. The high standard deviation of total household expenditure, household net income and household net asset indicate that there are some differences in consumption, income and wealth among different households.

**Table 1:** Descriptive statistics of the variables.

VARIABLES	OBSERVATIONS	AVERAGE	STANDAR VATIONS	MINIMUM	MAXIMUM	DECLARATIONS
**Total household consumption**	23,464	10.686	0.397	9.864	12.025	
**Gini coefficient**	23,464	0.489	0.043	0.154	0.598	
**Household debt with lag one period**	23,464	0.134	0.069	0	0.514	
**Net household income**	17,596	10.124	0.067	0.018	0.514	
**Total household assets**	23,464	12.589	0.617	11.399	14.812	
**Gender**	23,464	0.606	0.489	0	1	1 for male, 0 for female
**Age**	23,464	50.656	13.059	16	95	
**Age squared**	23,464	2566.030	170.537	256	9025	
**Education level**	23,464	7.236	4.520	0	19	
**Relationship**	23,464	0.884	0.320	0	1	1 for married, 0 for others
**Health status**	23,464	2.871	1.302	1	5	1 is very healthy, 5 is unhealthy
**Pop**	23,464	3.774	1.752	1	26	Family size
**Labor**	23,464	0.027	0.161	0	1	1 for unmarried adult male, 0 for others.

Note: Total household consumption, net household income, and total household assets in the table are logarithmic values, and total household consumption, household debt, net household income, and total household assets are taken as regional household averages.

**Fig 3:**
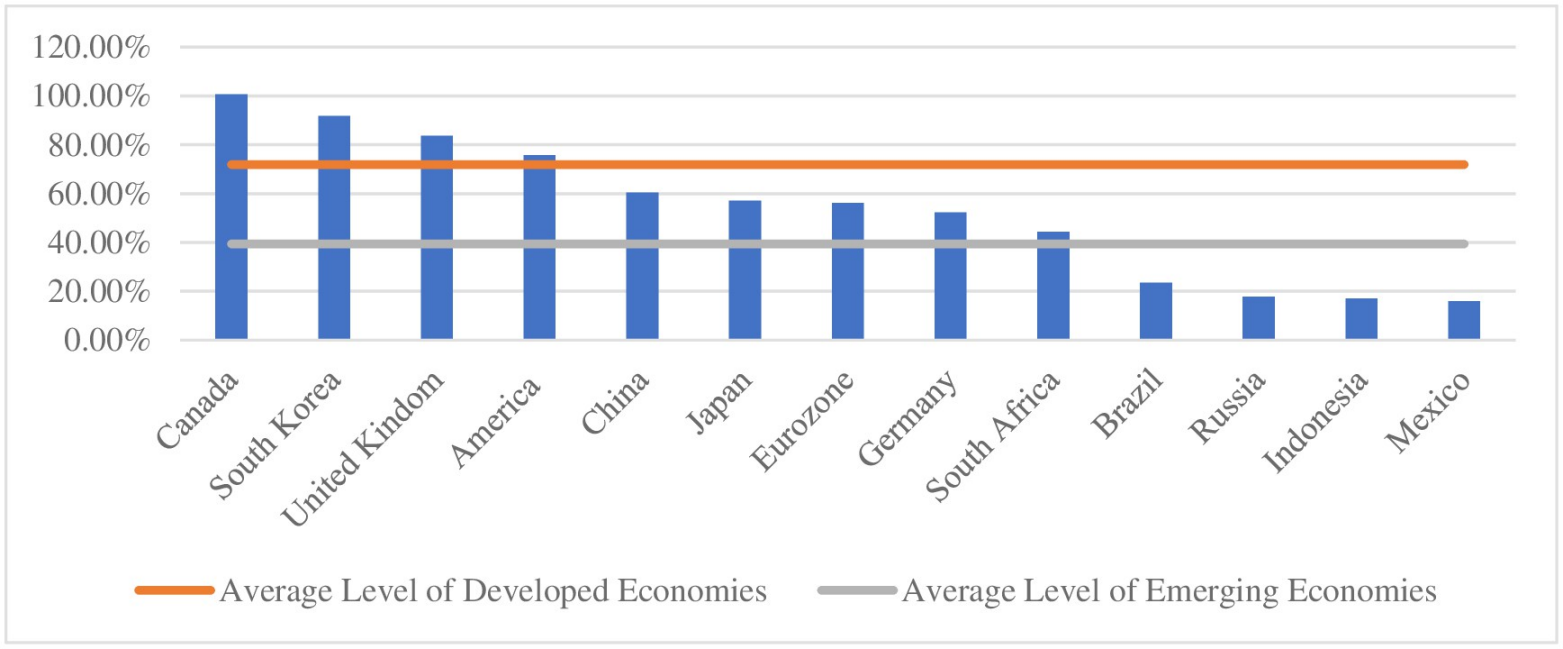
International comparison of residents’ leverage ratio.

In 2018, China’s resident leverage ratio is 60.5%, which is one of the highest leverage ratios among emerging market countries. The leverage ratio of residents in emerging market countries is generally low, with an average level of only 39.4%. Compared with developed countries, although China’s resident leverage ratio is lower than that of major countries such as the United States and Britain, it is higher than most of the EU member states. In addition, China’s residential leverage ratio is close to the international alert level. According to the IMF report, residential sector debt can affect the country’s medium-term economic growth when it exceeds 30% of GDP, while exceeding 65% can affect financial stability. The data of China comes from the survey and Statistics Department of the people’s Bank of China, and the data of other countries come from BIS (See Fig 3).

**Fig 4:**
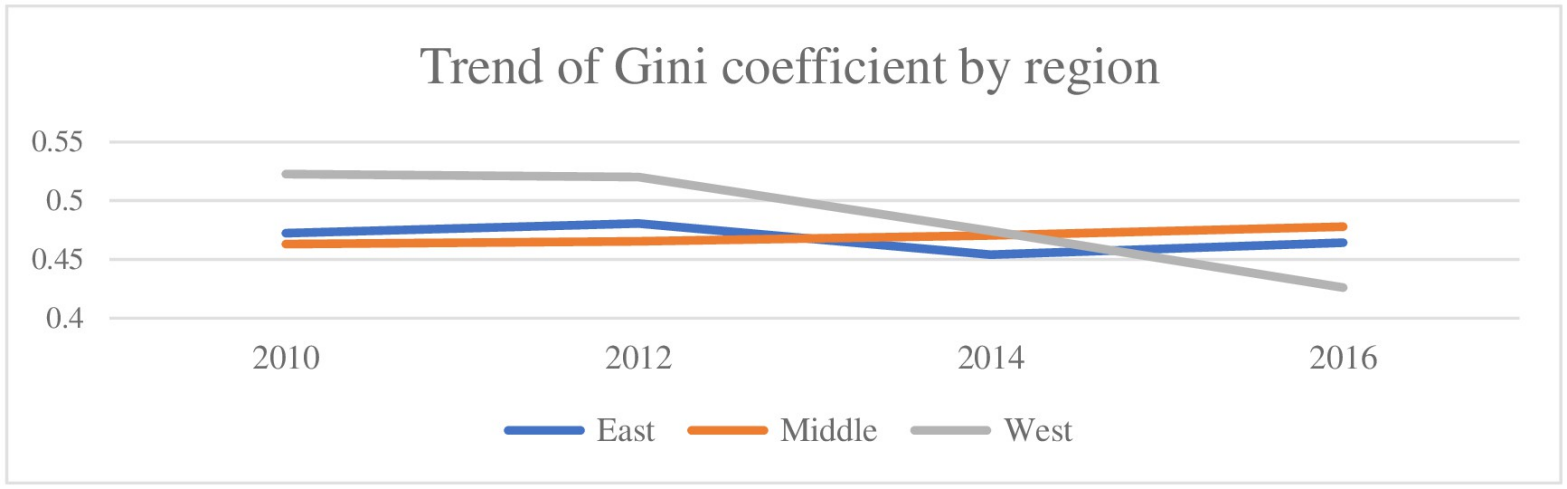
The trend of Gini coefficient in east, middle and west regions. Source: CFPS 2010–2016 database.

In addition, according to the trend chart of Gini coefficients in the eastern, central and western regions from 2010 to 2016 (see Fig 4), it is found that the Gini coefficient in the eastern region first increased, then decreased and then rose again, while the movement of the Gini coefficient of the central region was generally stable and rising, and the Gini coefficient of the western region decreased significantly. Due to the good geographic location, reasonable economic structure, adequate human capital and relatively more financial support, the Gini coefficient of the eastern region had steadily increased in the economic growth along with the steady increased in household income of all strata, making the Gini coefficient of the eastern region decrease slightly from 2012 to 2014, and increased slightly after 2014 but had been in a relatively stable state overall. The Gini coefficient of the central region had been rising slowly, and the difference with the Gini coefficient of the eastern region had remained almost within 0.2. However, the Gini coefficient of the western region declined significantly after 2012 and was lower than the income inequality of the central and eastern regions after 2014, probably because the state implemented a series of policies favoring the development during this period and increased investment in the western region, thus driving the local economic development and making the Gini coefficient of the western region decline faster. Table 2 shows the Gini coefficients of selected regions from 2010 to 2016.

**Table 2:** Gini coefficients for selected provinces.

Year	Beijing	Hebei	Shanghai	Guangdong	Shanxi	JiangxiI	Henan	Guangxi	Chongqing	Yunnan	Gansu
**2010**	0.418	0.512	0.443	0.536	0.509	0.429	0.452	0.530	0.554	0.461	0.564
**2012**	0.409	0.487	0.478	0.467	0.486	0.459	0.484	0.544	0.471	0.532	0.489
**2014**	0.480	0.468	0.389	0.484	0.499	0.471	0.465	0.543	0.487	0.511	0.493
**2016**	0.409	0.499	0.361	0.534	0.479	0.452	0.489	0.513	0.458	0.503	0.512

Source: CFPS 2010–2016 database.

## Empirical analysis

### Empirical results of the impact of income inequality and household debt on total household expenditure

Using Stata14 statistical software, we estimated models (1) and (2), where columns 2,4, 6 show the regression results for model (1) and columns 3, 5, 7 show the regression results for model (2). Based on the robust Hausman test, we use robust fixed-effects model, and at the same time, we consider time effects in the fixed-effects model to solve endogenous problems that may change over time. Therefore, we define it as two-way fixed effects model (FE_TW), which considering that both time and region differences may have an impact on the results. Meanwhile, in this paper, control variables will be gradually added to the regression to verify the robustness of the results. FE_robust1 is a two-way fixed effect model contains major target variables and the head of household information control variables, which exclude family information control variables such as income and assets. And FE_robust2 is a fixed model without considering time trend items.

From the results columns 2,4 and 6 of Table 3, we can see the Gini coefficient is almost negative and significant, indicating that the increase of income inequality has a significant negative effect on residents’ consumption. But household debt always has a significant promotion effect on residents’ consumption, suggesting that an appropriate increase in indebtedness of Chinese households can effectively promote higher consumption and increase domestic demand. Columns 3, 5 and 7 of Table 3 respectively show the addition of the cross (*Gini*_jt_*lev_*jt*−1_) between income inequality and household debt to model (2). We can see that when the cross term is included, the coefficient of the interaction term is significant indicating that income inequity does have a moderating effect on household debt affecting residential consumption. Moreover, the interaction term coefficient *κ*_1_ of income inequality and household debt is significantly positive, indicating that the rising household debt gives more households a chance to boost their own consumption, easing the crowding out effect of income inequality. The possible reason is that the increase in residential household debt is structural in nature, the household savings rate of low-income households is generally low, and the increase in household debt significantly reduces the propensity to consume of the low-income group, thus reinforcing the negative effect of income inequality on consumption. Meanwhile, because both household assets and household debt have positive effects on total household expenditure, i.e., as household leverage rises, the rise in the value of household assets contributes more substantially to the increase in household expenditure and further validates the existence of the wealth effect. In addition, the coefficient of net household income is also positive and significant, indicating that the increase in household net income has a positive effect on total household expenditure, so the income effect also exists, proving that the development of consumer finance in China is still at a relatively low level and the development of inclusive finance can promote residents’ consumption. In addition, we also observe that the two-way fixed effect model (FE-TW) regression have a higher R2 among the three regression models, indicating that the models fit better after controlling for time and province.

**Table 3:** Income inequality and household debt on total household consumption.

VATIABLES	HOUSEHOLD CONSUMPTION					
	FE_robust1		FE_robust2		FE-TW	
	(1)	(2)	(3)	(4)	(5)	(6)
**Gini**	−0.808***	−0.752***	−0.063*	−0.037	−0.364***	−0.339***
	(0.0409)	(0.0431)	(0.0342)	(0.0350)	(0.0268)	(0.0273)
***Lev***_**t−1**_	0.207***	0.206***	0.371***	0.377***	0.253***	0.253***
	(0.0291)	(0.0242)	(0.0234)	(0.0228)	(0.0234)	(0.0204)
Gini*Lev_**t−1**_		5.859***		2.659***		3.780***
		(0.5800)		(0.4750)		(0.4650)
**Ln(income)**			1.121***	1.117***	0.485***	0.475***
			(0.0137)	(0.0139)	(0.0169)	(0.0180)
**Ln(asset)**			0.247***	0.245***	0.0284***	0.0252***
			(0.0075)	(0.0075)	(0.0052)	(0.0055)
**_Cons**	11.090***	11.060***	−4.319***	−4.271***	5.326***	5.460***
	(0.0324)	(0.0769)	(0.1410)	(0.1430)	(0.2040)	(0.2180)
**Year FE**	Yes	Yes	No	No	Yes	Yes
**Province FE**	Yes	Yes	Yes	Yes	Yes	Yes
***R*** ^**2**^ **_*adjust***	0.8923	0.8947	0.7739	0.7744	0.9150	0.9160
**Sample size**	17598	17598	17598	17598	17598	17598

Note:

***, **, and * indicate significant at the 1%, 5%, and 10% levels of significance, respectively.

Due to space constraints, the results of correlation tests for other control variables are not presented in the paper and can be obtained by contacting the authors if you are interested.

### Expanded analysis

#### Estimation results of regional differences

Due to the uneven economic development in China and the prevalence of regional differences, many domestic and international scholars have studied income inequality and regional differences in household finance in China. Demurger et al. [35] found that past literature had overestimated the extent of income inequality in China, and that widening regional disparities had increased income inequality. Goh et al. [36] used CHNS (China Health and Nutrition Survey) to increase income inequality in eight provinces and autonomous regions from 1998 to 2004, they found that there were disparities in income inequality between urban and rural areas in China. Wen [37] analyzed the impact of inclusive finance on regional income inequality based on China’s provincial panel data, and the results showed that the development of inclusive finance helps to reduce regional and urban-rural income inequality, and this effect increases with the development of inclusive finance.

As seen in Fig 1, the degree of income inequality varies among the eastern, central and western regions, and there is a wide gap between the economic development and consumption capacity of each region. This paper use the two-way fixed effects (FE_TW) regression analyzes the impact of income inequality and household debt on consumption, and further discusses whether there is a regional difference in this impact (see the Table 4).

**Table 4:** Regional differences in the impact of income inequality and household debt on household consumption.

VARIABLES	HOUSEHOLD CONSUMPTION					
	Eastern		Central		Western	
	(1)	(2)	(3)	(4)	(5)	(6)
**Gini**	−0.477***	−0.392***	0.603***	0.569***	−1.830***	−2.144***
	(0.0586)	(0.0686)	(0.0833)	(0.1020)	(0.0781)	(0.0910)
***Lev***_***t*−1**_	0.099**	0.189***	0.329***	0.298***	0.351***	−0.106***
	(0.0504)	(0.0584)	(0.0678)	(0.0623)	(0.0187)	(0.0257)
Gin*Lev_***t*−1**_		1.914**		1.436		21.660***
		(0.7930)		(1.0580)		(1.2240)
**Ln(income)**	0.554***	0.551***	0.269***	0.264***	0.244***	0.304***
	(0.0454)	(0.0471)	(0.0249)	(0.0246)	(0.0329)	(0.0321)
**Ln(asset)**	−0.004	−0.003	−0.223***	−0.215***	0.531***	0.554***
	(0.0072)	(0.0073)	(0.0297)	(0.0272)	(0.0308)	(0.0326)
**_Cons**	5.054***	5.019	10.160***	10.130***	2.458***	1.768***
	(0.5800)	(0.5900)	(0.2820)	(0.2700)	(0.5580)	(0.5610)
**Year FE**	Yes	Yes	Yes	Yes	Yes	Yes
***R*** ^**2**^ **_*adjust***	0.9404	0.9407	0.9288	0.9289	0.9415	0.9530
**Sample size**	7263	7263	5403	5403	4932	4932

Note:

***, ** and * indicate significant at 1%, 5% and 10% significance levels, respectively.

According to the results of the model which can be seen in the Table 4, the increase of income inequality can promote the total household consumption in the central area, for the eastern and the western regions, the expansion of income inequality has a restraining effect on total household consumption. When the interaction term is not included, household debt has a positive effect on total consumption, which shows that appropriate increases in borrowing can help boost household consumption. When the cross term is included, in the eastern region, we can see that the household debts maintain positive and the cross term keeps positive significant, which indicating a reduction in income inequality will significantly enhance the positive effect of household debt on residents’ consumption. But in the central area, the cross term is not significant which indicate that income inequity has no significant effect on the association between household debt and household consumption. In the western region, however, the interaction coefficient becomes significantly positive and larger than that in the central and eastern regions. It shows that for the western region, appropriately increasing the borrowing level of residents and reducing the income gap can exponentially boost consumption growth in this region. For the relatively developed central and eastern regions, the impact of average household asset on total household consumption is negative, which may be due to the households in relatively developed areas are more inclined to acquire property wealth through mortgages, and although the total assets of households increase, the repayment pressure increases accordingly, thus squeezing out consumption. However, the influence of household income on household consumption is positive and significant, indicating that the increase of household income will help to promote consumption.

#### Results analysis of urban-rural differences

The long-term dualistic economic structure has led to large differences between urban and rural households in China in terms of household income, household debt, and household consumption, which in turn may have different effects on the association between household leverage and consumption for urban and rural residents. Therefore, this paper further analyzes the impact of income inequality and household debt on household consumption by dividing the sample into urban and rural households (see the Table 5).

**Table 5:** Urban-rural differences in income inequality and household debt on household consumption.

VARIABLES	HOUSEHOLD COMSUMPTION			
	Urban Residents		Rural Residents	
	(1)	(2)	(3)	(4)
**Gini**	−0.232***	−0.203***	−0.483***	−0.495***
	(0.0338)	(0.0340)	(0.0294)	(0.0299)
***Lev***_***t*−1**_	0.149***	0.193***	0.349***	0.230***
	(0.0309)	(0.0270)	(0.0327)	(0.0363)
Gini*Lev_***t*−1**_		3.245***		7.892**
		(0.660)		(1.003)
**Ln(income)**	0.435***	0.408***	0.312***	0.309***
	(0.0261)	(0.0305)	(0.0309)	(0.0314)
**Ln(asset)**	0.0365***	0.0307***	−0.0276***	−0.0265***
	(0.00887)	(0.0102)	(0.00800)	(0.00797)
**_Cons**	5.704***	6.041***	7.877***	7.910***
	(0.316)	(0.384)	(0.399)	(0.401)
**Year FE**	Yes	Yes	Yes	Yes
***R*** ^**2**^ **_*adjust***	0.9188	0.9198	0.9183	0.9203
**Sample size**	8031	8031	9463	9463

Note:

***, ** and * indicate significant at 1%, 5% and 10% significance levels, respectively.

As shown in the Table 5, regression analysis models (1) and (2) by two-way fixed effect (FE_TW) model we can see that the regression result is approximately the same as that of the total sample. Income inequality has a negative impact on total household expenditure, and the increase in household debt contributes to total household expenditure, and the interaction term coefficient is significantly positive which indicating that the rising household debt gives more households a chance to boost their own consumption, easing the crowding out effect of income inequality. Compared with the urban household expenditure, the impact of income inequality and household debt on the rural household expenditure is greater, especially the Gini coefficient, which estimated value of the coefficient is almost 2 times of that in urban areas, indicating that the restraining effect of income inequity in rural areas on consumption is far more than that in urban areas. So, reducing the level of income inequity in rural areas can effectively stimulate residents’ consumption. The above findings prove that, in order to increase residents’ consumption and promote balanced development in urban and rural areas, the government not only needs to continuously introduce more beneficial policies in rural areas, but also should make use of the leverage of consumer finance and vigorously promote the innovation of rural financial instruments.

### Robustness test

Because Gini coefficient is only one of the indicators to measure income inequality, its calculation method also has some limitations. Moreover, since the Gini coefficient in this paper is calculated based on the net income per capita of households in the CFPS database, the business income and property income in the net income of households might be influenced by economic cycles and other factors, and the uncertainty of transfer income is also greater, the stability of household net income will also have a certain impact. In addition, the Gini coefficient is more sensitive to the income of families in the middle of income inequality, which reduces the credibility of the Gini coefficient to a certain extent.

Therefore, in order to verify the findings of this paper, we also use other measures in the Table 6, such as the provincial Theil index and the Mean Logarithmic Deviation (MLD) index as proxy variables for income inequality to test the robustness of the empirical results. The results show that the main conclusions are basically consistent with those mentioned above, so the main conclusions of this paper are proved to be robust.

**Table 6:** Regression results of other measures of income inequality.

**VARIABLES**	**HOUSEHOLD CONSMPTION**					
	**FE_robust1**		**FE_robust2**		**FE-TW**	
	(1)	(2)	(3)	(4)	(5)	(6)
***Lev***_***t*−1**_	0.213***	0.222***	0.386***	0.384***	0.254***	0.257***
	(0.0285)	(0.0252)	(0.0240)	(0.0247)	(0.0230)	(0.0216)
**Theil index**	−0.175***	−0.169***	−0.082***	−0.083***	−0.096***	−0.095***
	(0.0111)	(0.0106)	(0.0106)	(0.0107)	(0.0082)	(0.0081)
Theil * *Lev*_***t*−1**_		1.954***		−0.125		0.818***
		(0.1790)		(0.1880)		(0.1500)
**Ln(income)**			1.122***	1.122***	0.512***	0.505***
			(0.0142)	(0.0144)	(0.0169)	(0.0178)
**Ln(asset)**			0.246***	0.246***	0.024***	0.022***
			(0.0074)	(0.0074)	(0.0054)	(0.0056)
**_Cons**	10.780***	10.780***	−4.321***	−4.328***	4.948***	5.053***
	(0.0238)	(0.0237)	(0.1500)	(0.1540)	(0.2040)	(0.217)
**Year FE**	Yes	Yes	No	No	Yes	Yes
**Province FE**	Yes	Yes	Yes	Yes	Yes	Yes
***R*** ^**2**^ **_*adjust***	0.8876	0.8895	0.7748	0.7748	0.9145	0.9148
**Sample size**	17598	17598	17598	17598	17598	17598
**VARIABLES**	**HOUSEHOLD CONSMPTION**					
	**FE_robust1**		**FE_robust2**		**FE-TW**	
	(1)	(2)	(3)	(4)	(5)	(6)
***Lev***_***t*−1**_	0.265***	0.210***	0.369***	0.372***	0.280***	0.245***
	(0.0297)	(0.0246)	(0.0231)	(0.0239)	(0.0237)	(0.0198)
**MLD**	−0.313***	−0.319***	−0.010	−0.011	−0.111***	−0.121***
	(0.0168)	(0.0164)	(0.0150)	(0.0151)	(0.0120)	(0.0117)
MLD*Lev_***t*−1**_		2.452***		−0.409**		1.648***
		(0.1910)		(0.1910)		(0.1590)
**Ln(income)**			1.123***	1.121***	0.493***	0.485***
			(0.0138)	(0.0138)	(0.0176)	(0.0184)
**Ln(asset)**			0.246***	0.247***	0.027***	0.020***
			(0.0074)	(0.0075)	(0.0053)	(0.0057)
**_Cons**	10.860***	10.870***	−4.356***	−4.350***	5.129***	5.321***
	(0.0251)	(0.0249)	(0.1430)	(0.1430)	(0.210)	(0.2250)
**Year FE**	Yes	Yes	No	No	Yes	Yes
**Province FE**	Yes	Yes	Yes	Yes	Yes	Yes
***R*** ^**2**^ **_*adjust***	0.8914	0.8944	0.7738	0.7739	0.9156	0.9156
**Sample size**	17598	17598	17598	17598	17598	17598

Note: Other control variables are consistent with Table 2, and regression results for specific control variables are not presented for space reasons.

***, **, and * indicate significant at the 1%, 5%, and 10% levels of significance, respectively, and t-values calculated with robust standard errors are reported in parentheses.

## Conclusions and policy recommendations

Based on the existing literature review, this paper uses CFPS (China Family Panel Studies) database to study the direct and indirect impacts of income inequality and household debt on household consumption, as well as the differences of these impacts among different regions. The empirical results show that, firstly, income inequality has a significant negative effect on residential household consumption, while household debt has a significant positive effect on household consumption. In the two-way fixed effects model (controlling for province and year), after adding interactive item (*Gini*_jt_*lev_*jt*_), the coefficient of the cross term is significantly positive at the 1% level, it suggests that the rising household debt gives more households a chance to boost their own consumption, easing the crowding out effect of income inequality. Secondly, different from the total samples analysis, the increase of income inequality promotes the total household consumption in the central areas where the economy is not very developed, but the cross term is not significant which indicate that income inequity has no significant effect on the association between household debt and household consumption in the midland. For the eastern and western regions, the result is as same as total samples analysis. However, in the western region, the interaction coefficient becomes significantly positive and larger than central and eastern areas. It shows that for the western region, appropriately increasing the borrowing level of residents and reducing the income gap can exponentially boost consumption growth in this region. Thirdly, compared with the urban household expenditure, the impact of income inequality and household debt on the rural household expenditure is greater, especially the Gini coefficient, which estimated value of the coefficient is almost 2 times of that in urban areas, indicating that the restraining effect of income inequity in rural areas on consumption is far more than that in urban areas. At the same time, from the perspective of rural household consumption, household debt has a greater impact on rural household expenditure. The above findings prove that, in order to increase residents’ consumption and promote balanced development in urban and rural areas, the government not only needs to continuously introduce more beneficial policies in rural areas, but also should make use of the leverage of consumer finance and vigorously promote the innovation of rural financial instruments.

This paper studies the impact of income inequality and household debt on household consumption from a micro perspective, providing a new perspective to explain the current consumption problems in China. Compared with the existing literature research, the contribution of this paper may be reflected in the following aspects. Firstly, we construct a balanced panel data model that links the household consumption to income inequality and household debt. In this model, Gini coefficient of income is used as the independent variable to measure income inequality, and the factors such as household debt ratio, household net income and household worth are considered to explore the impact of the interaction between income inequality and household debt on total household consumption. And do a further study on the consumer finance which has enriched the research in this field. Secondly, using Theil index and the Mean Logarithmic Deviation (MLD) index instead of Gini coefficient as the alternative variable to measure income inequality to test the robustness of the empirical results, which makes the estimation results more reliable. Finally, by further analyzing the difference between consumer finance in different regions, expanding domestic demand and stimulating residents’ consumption, it is helpful to improve China’s consumption quality and unreasonable economic structure, to provide theoretical basis and policy suggestions for realizing consumption upgrading and sustainable development of consumption.

In view of the impact of COVID-19, the income of some residents decreased significantly, and uncertainty about future expectations makes households more inclined to save rather than spend. According to financial data released by the Central bank, household deposits increased by 6.47 trillion yuan in the first quarter, an increase of 6.6% over 2019. There are more than half of households (50.2%) chose to increase savings and reduce consumption, 40.4% of households maintained the current situation, and only 9.4% of households reduced savings and increased consumption. In addition, the willingness to save is much higher for low-income groups than for high-income groups, especially for households whose income decreases much during the epidemic, their willingness to save is more intense, and the debt consumption is further reduced. However, it is not possible to make further judgments because the household data are not updated, and further research is needed.
